# Effect of Synthesis Conditions on CuO-NiO Nanocomposites Synthesized via Saponin-Green/Microwave Assisted-Hydrothermal Method

**DOI:** 10.3390/nano14030308

**Published:** 2024-02-03

**Authors:** Amnah Al-Yunus, Wafa Al-Arjan, Hassan Traboulsi, Robson Schuarca, Paul Chando, Ian D. Hosein, Manal Hessien

**Affiliations:** 1 Department of Chemistry, College of Science, King Faisal University, P.O. Box 400, Alahsa 31982, Saudi Arabia; 2 Department of Chemistry, Champlain College, 900 Riverside Drive, St-Lambert, QC J4P 3P2, Canada; 3 Department of Biomedical and Chemical Engineering, College of Engineering and Computer Science, Syracuse University, 339 Link Hall, Syracuse, NY 13244, USA

**Keywords:** CuO-NiO nanocomposites, saponin, green hydrothermal-assisted microwave, microstructure

## Abstract

This work presents the synthesis of CuO-NiO nanocomposites under different synthesis conditions. Nanocomposites were synthesized by merging a green synthesis process with a microwave-assisted hydrothermal method. The synthesis conditions were as follows: concentration of the metal precursors (0.05, 0.1, and 0.2 M), pH (9, 10, and 11), synthesis temperature (150 °C, 200 °C, and 250 °C), microwave treatment time (15, 30, and 45 min), and extract concentration (20 and 40 mL of 1 g saponin/10 mL water, and 30 mL of 2 g saponin/10 mL water). The phases and crystallite sizes of the calcined nanocomposites were characterized using XRD and band gap via UV-Vis spectroscopy, and their morphologies were investigated using SEM and TEM. The XRD results confirmed the formation of a face-centered cubic phase for nickel oxide, while copper oxide has a monoclinic phase. The calculated crystallite size was in the range of 29–39 nm. The direct band gaps of the samples prepared in this work were in the range of 2.39–3.17 eV.

## 1. Introduction

Owing to their outstanding physical and chemical capabilities, nanomaterials have received significant attention. Nanomaterials exhibit diverse arrays of shapes and structures. With at least one dimension in the nanoscale, nanomaterials can take on various geometrical forms, such as nanowires, nanotubes, nanorods, nanoflowers, and nanosheets. Metal oxides are recognized for their abundance and non-harmful nature. Nanoscale metal oxides have expansive surface areas and exhibit robust thermal and chemical stability. CuO, NiO, and ZnO have received considerable attention [1,2,3]. Nanosized metal oxides have numerous applications, including supercapacitors, hydrogen generation, solar cells, sensors, and photocatalytic pollutant destruction [3].

Nickel oxide is a wide bandgap semiconductor of the p-type variety, presenting a bandgap that spans from 3.4 eV to 4.0 eV. It has practical applications in the development of antibacterial coatings [4], catalysts, gas sensors, cathodes, dye-sensitized solar cells, antiferromagnetic layers, and electrochromic devices [5,6,7]. Copper oxide is a narrow-band semiconductor of the p-type variety with a bandgap that spans from 1.2 to 2 eV [7]. Copper oxide is known for its cost-effectiveness, lack of toxicity, and optical and electrical properties. It has applications in solar cells, magnetic storage devices, and gas sensors [7].

Nanocomposites of metal oxides provide better characteristics by combining the individual characteristics of metal oxides to form nanocomposites with tunable properties, synergistic effects, and enhanced catalytic activity [3]. It has been reported that combining CuO with NiO results in improved crystallinity, antifungal properties, and optical and luminescent properties [7].

Numerous techniques are available for the synthesis of metal oxides, either in the form of pure metal oxides or nanocomposites. These techniques involve precipitation [8,9], vapor phase growth [6], electrospinning [5], sol–gel combustion [1], hydrothermal methods [10,11], solution combustion synthesis [11], sonochemical routes [2,12,13,14], and solvothermal methods [15]. The hydrothermal approach is a simple, affordable, and quick method for synthesizing at low temperatures. The microwave–hydrothermal process combines the benefits of the hydrothermal technique (i.e., reduced reaction time and lowered temperature of synthesis) with the benefits of the microwave technique (i.e., energy efficiency, contactless method, and scalability) [10,11,16]. Green synthesis employs plant extracts as natural capping agents instead of synthetic surfactants. This approach offers advantages such as simplicity, environmental friendliness, rapid reactions, low calcination temperatures, and easy scalability.

Nanomaterials undergo aggregation, which results in a decrease in surface area. Capping agents are used to decrease aggregation and direct growth, which results in a controlled size and morphology [17]. Surfactants, polymers, and natural compounds are examples of capping agents [17]. Natural compounds can be extracted from various biological sources, such as leaves, fruits, fruit peels, roots, and seeds, and from green substrates such as glucose, vitamins, fungi, and natural polymers [17]. Artificial surfactants trigger environmental and toxic concerns due to their emission of cancer-causing toxins, which is why more interest in natural surfactants is emerging [18]. Saponin has the chemical formula C_52_H_84_O_21_.2H_2_O and is an example of a natural surfactant. This natural surfactant can be extracted from a fruit called Indian soapberry, found on a soapnut tree (Sapindus mukorossi) [19,20,21]. Soapnuts have various names around the world, such as “ritha, reetha, soapnut, washnut, soapberry, sapindus trifoliatus and sapindus mukorossi” [21]. Soapnuts are recognized for their commercial use in detergents, cosmetics, and other products. Additionally, these nuts possess medicinal properties, including anti-inflammatory and antimicrobial properties [22,23]. Because of their simplicity and environmental friendliness, the utilization of botanical sources for the environmentally friendly synthesis of metal oxide nanoparticles is becoming increasingly prominent. This is primarily because of the considerable potential of plants to function as both reducing and capping agents, a characteristic attributed to their wide array of phytochemicals and antioxidant compounds [24].

The application of soapnut extract as a capping agent in the preparation of metal oxides is limited. T. Saikia et al. prepared copper oxide nanosized with soapnut extract integrated with hydrothermal method. They examined the effect of extract concentration on the size of the synthesized copper oxide. SEM results indicated that the concentration of the extract increased as the particle size decreased [25]. V. Jassal et al. prepared manganese oxide using soapnut extract. They applied manganese oxide to the oxidation of aromatic amines [26]. M. Hessien et al. prepared ZnO-Bi_2_O_3_ and La_2_O_3_-Fe_2_O_3_-Bi_2_O_3_ using soapnut green synthesis-assisted microwave–hydrothermal method and studied the synthesized composites in radioactive shielding [27,28]. B. Debnath and colleagues prepared silver nanoparticles using soapnut extract as a reducing agent [29]. Au nanoparticles were prepared by V. Reddy and colleagues with the assistance of soapnut extract [22]. 

D. Nzilu et al. prepared copper oxide nanoparticles using an aqueous extract of Parthenium hysterophorus. The average crystallite size was approximately 32 nm, and the degradation efficiency of rifampicin was approximately 98% [30]. R. Kumar et al. prepared NiO and Eu-doped NiO using a microwave–hydrothermal method, and their results indicated the production of crystalline nickel oxide with a flake morphology [31]. G. Anand et al. utilized the microwave combustion method in the presence of green extract derived from either Moringa oleifera or Punica granatum to synthesize CuO nanoparticles [32].

CuO-NiO nanocomposites have been prepared via various synthesis methods, such as hydrothermal method [33,34,35], green-assisted hydrothermal methods [36], combustion methods [37], chemical precipitation [38,39], electrospinning [40], decomposition of metal–organic frameworks [41], and sol–gel [42]. H.D. Weldekirstos and colleagues synthesized CuO-NiO nanocomposites via coprecipitation, utilizing CTAB as a structure-directing agent, and subsequently subjected them to calcination at 450 °C for 3 h [38]. T. Noor et al. prepared CuO-NiO metal oxide framework-reduced graphite oxide nanocomposites using a hydrothermal method and applied it in electrocatalysis in a fuel cell with a current density of ~437 mA/cm^2^ [43]. P. Muhambihi et al. prepared CuO-NiO, ZnO-NiO, and CuO-ZnO nanocomposites using a simple coprecipitation method. The CuO-NiO nanocomposite exhibited a p-p isotype heterojunction with better electronic interactions, while the other two composites showed p–n heterojunctions. The CuO-NiO nanocomposite showed better dye degradation [44]. 

L. Arun et al. prepared CuO-NiO nanocomposites using green synthesis with the aid of an extract of Azadirachta indica and applied it in the decomposition of MB dye with ~99% [45]. M. Hassanpour et al. prepared CuO-NiO nanocomposite via microwave synthesis for a short time (15–30 min.) in the presence of Tween 20. The samples showed magnetic properties along with photocatalytic degradation of the MO dye [10]. M. Mwhmadi et al. used olive oil as a structure-directing agent along with microwave synthesis for a few minutes for CuO nanoparticles and NiO nanoparticles, separately without composite formation. They studied the effects of microwave power, microwave time, and olive oil volume. They found that all parameters affected the agglomeration of nanoparticles differently [46].

To the best of our knowledge, the preparation of NiO-CuO nanocomposites using saponin extract as a capping agent has not been reported. Furthermore, no data on mixing saponin extract with the microwave-assisted hydrothermal technique in the synthesis of CuO-NiO have been reported. In this paper, we present an effective method for synthesizing CuO-NiO nanocomposite powders under various conditions. Thus, a novel green synthesis based on saponin extract was integrated with a microwave-assisted hydrothermal method. X-ray diffraction (XRD), infrared spectroscopy (FTIR), UV-Vis spectroscopy, scanning electron microscopy (SEM), and transmission electron microscopy (TEM) were used to examine the synthesized samples.

## 2. Materials and Methods

### 2.1. Materials

Copper nitrate trihydrate (Cu(NO_3_)_2_·6H_2_O), nickel nitrate hexahydrate (Ni(NO_3_)_2_·6H_2_O), and ammonium hydroxide solution 30% were purchased from Sigma-Aldrich, Shanghai, China. The chemical compounds used in this study were of analytical grade, and double-distilled water was used to prepare the solutions. The soapnuts, which were sun-dried, de-seeded soap berries, were purchased from NatureOli Beautiful in Peoria, AZ, USA, and were USDA-certified organic.

### 2.2. Saponin Extraction

The soapnuts were pulverized into a fine powder using a common household grinder. Subsequently, deionized water was used to dissolve the soapnut powder at a ratio of 1 g of soapnut powder in 10 mL of water. The resulting mixture was stirred for 3 h at 60 °C. Subsequently, the extract was separated using Whatman filter paper and stored at 4 Â °C in a refrigerator. This filtrate was designated as the “Saponin extract”. 

### 2.3. Microwave-Assisted Hydrothermal Synthesis

**Scheme 1:**
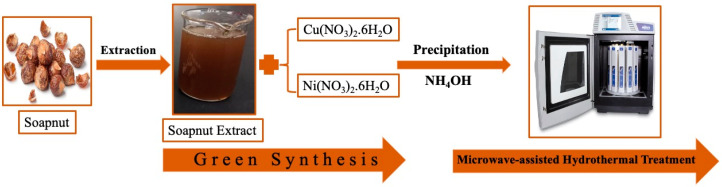
Synthetic method of CuO-NiO nanocomposites.

The specified quantities of nickel salt and copper salt were weighed and used to create separate stock solutions, each with a concentration of 0.1 M. Details regarding sample compositions and names can be found in Table 1. The process involved mixing 50 mL of each nickel and copper stock solution, followed by the addition of 20 mL of a natural surfactant extract. To adjust the pH of the mixture, NH_4_OH solution was employed. Moreover, the NH_4_OH solution was gradually poured in from a burette at a pace of 1 mL per minute while continuously stirring the mixture. The pH of the solution was measured using an Orion 2 Star pH meter until the desired pH level was reached. A representation of this synthetic method is shown in Scheme 1. Subsequently, the obtained hydroxide precipitate was transferred from the container to a Teflon vessel and subjected to microwave heating at a specified temperature for a predetermined duration. The microwave used was Titan MPS microwave, Perkin Elmer, with a maximum power of 2700 W and 10 Teflon vessels (75 mL each).

Subsequently, the sample was removed from the microwave and transferred to a beaker after the liquid was decanted. To process the sample further, a Power Sonic 405 system was employed for sonication, which was conducted for 90 min using 50 mL of distilled water. Following this step, the solid oxide was allowed to precipitate naturally under the influence of gravity for 12 h, after which the liquid was decanted, and 20 mL of ethanol was introduced. The sonication and precipitation processes were repeated, and the resulting sample was dried in an oven at 100 °C for 12 h. Finally, the samples were calcined at 500 °C for 2 h to eliminate any remaining organic residues. The calcination was done in a Sanwood Box-type Resistance Furnace, 2.5 kW.

### 2.4. Powder Characterization

FTIR spectra of the synthesized metal oxide nanocomposite samples were acquired using a Cary 630 FT-IR spectrophotometer. To identify the crystalline phases within the metal oxide nanocomposites, XRD analysis was performed using a Bruker D8 X-ray diffractometer employing Ni-filtered Cu-Kα radiation and a graphite monochromator, producing X-rays with a wavelength of 1.54060 Å at 35 kV and 25 mA. The analysis involved scanning over a glancing angle range of 10°–60° in increments of 0.02°, with an accuracy level of ≤0.001°. The crystallite size was determined using the Scherrer Formula (1).

(1) $$D=\frac{0.9\;\lambda }{\beta cos\theta }$$

In the provided formula, *D* represents the average size of the well-structured (crystalline) domains in nanometers, *λ* represents the wavelength of the X-rays in nanometers, *β* stands for the line broadening at the half-width maximum intensity (FWHM), and *θ* represents the Bragg angle in degrees.

UV-Vis spectra were recorded on a UV-Vis spectrophotometer. The Direct band gap energy was calculated from the Tauc relation in Equation (2) [47]:

(2) $${\left(\alpha h\nu \right)}^{n\;}=A(h\nu -E_{g}),$$

where E_g_ is the direct band gap if *n* = 2, h is Blanck’s constant, and *υ* is the frequency. Plotting *hυ* against (α *hυ*)^2^ results in a figure where the intersection with the x-axis gives the value of the direct band gap.

To further examine the surface morphology and structure, a Scanning Electron Microscope (SEM) with a Philips XL30 was used, with an accelerating voltage of 30 kV and magnification capabilities of up to 400,000×. High-resolution Transmission Electron Microscopy (TEM) was performed using a JEOL JEM-1011 Transmission Electron Microscope. A tiny volume of colloidal suspension is carefully dispensed onto a TEM grid and left to dry at room temperature. Once the medium has evaporated, the grid can be examined directly using TEM.

## 3. Results and Discussion

### 3.1. FTIR Characterization

**Figure 1:**
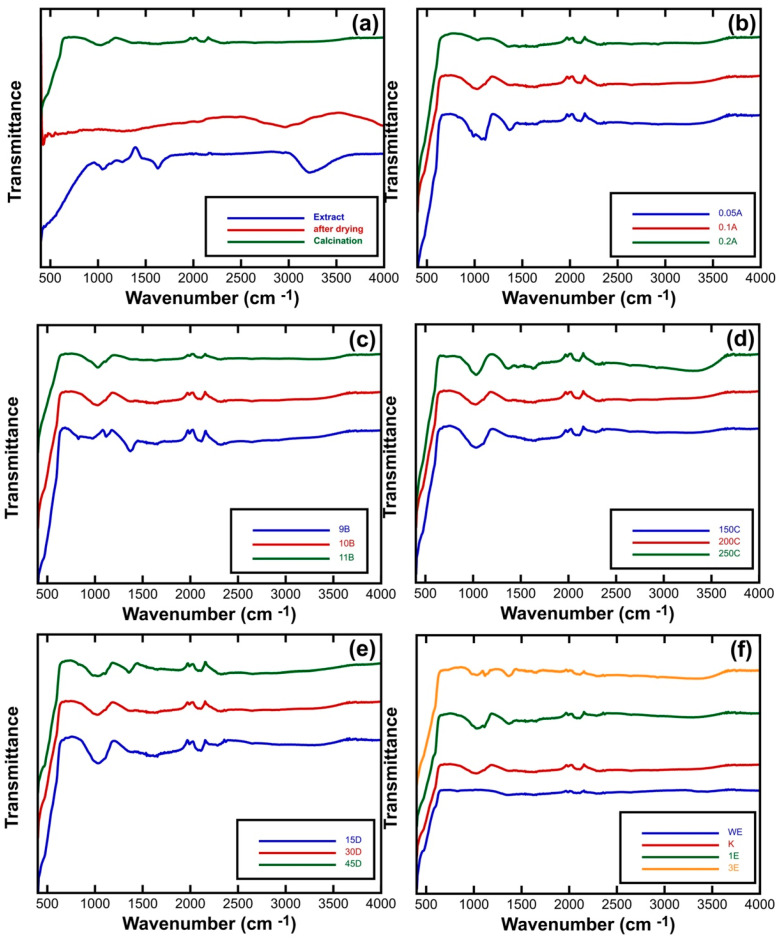
FTIR spectra of CuO-NiO nanocomposites (**a**) synthesis steps, (**b**) precursor concentration, (**c**) pH, (**d**) temperature, (**e**) time, and (**f**) extract concentration.

Figure 1a shows the FTIR spectra of the saponin extract and sample 0.1A after drying and calcination for 2 h at 500 °C. The FTIR spectrum of the saponin extract showed broad bands at 2900–3500 cm^−1^ for CH_2_ and hydroxyl groups, at 1630 cm^−1^ for the carbonyl group, and at 1050 cm^−1^ for the C-O-C group [19,21,48,49,50]. The matching of the FTIR bands in this work with the bands reported in the literature confirms the presence of saponin in the aqueous extract. After drying, the sample showed a hydroxyl group at approximately 3000 cm^−1^ for adsorbed water on the surface of the powder. After calcination, the hydroxyl group disappeared, and a deep band between 400 and 620 cm^−1^ appeared, which was attributed to the Ni-O and Cu-O bonds [47]. 

Figure 1b–f shows the FTIR spectra of calcined CuO-NiO nanocomposites after calcination at 500 °C, where the samples were prepared at different precursor concentrations, pH, temperature, time, and extract concentrations. All samples showed a wide band (3000–3500 cm^−1^) corresponding to the stretching vibration of adsorbed water molecules on the surface of the calcined powders. The observed band at 1050 cm^−1^ may be attributed to adsorbed CO_2_ from the ambient atmosphere. The band observed at 1360 cm^−1^ may be attributed to the NO_3_^–^ group from the precursors. All samples showed characteristic bands of Cu-O and Ni-O between 400 and 620 cm^−1^, confirming the formation of metal oxides after calcination at 500 °C [34,45,47,51,52,53].

### 3.2. XRD Characterization

**Figure 2:**
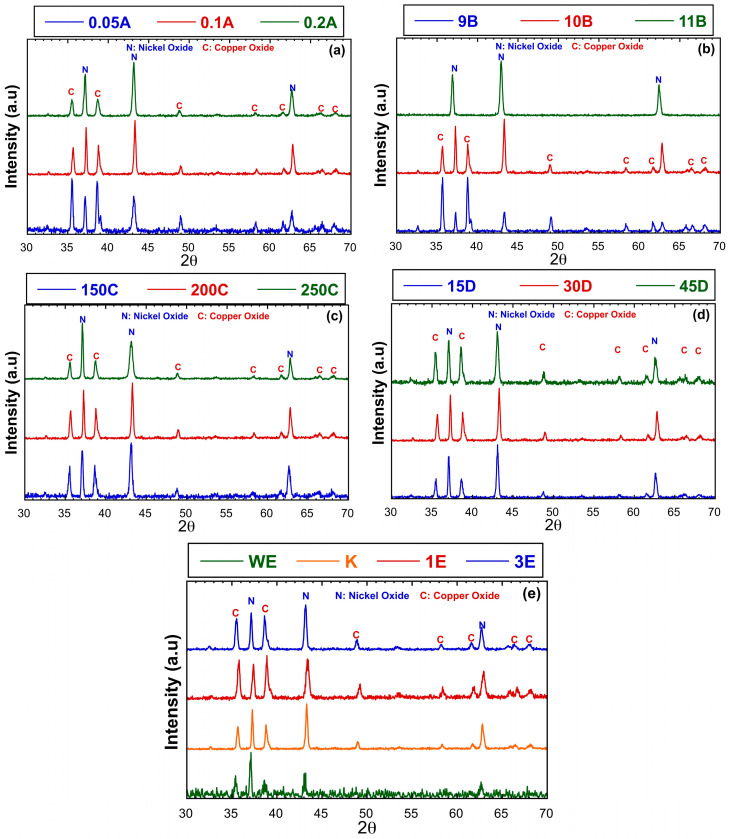
XRD diagrams of CuO-NiO nanocomposites: (**a**) precursors concentrations, (**b**) pH, (**c**) temperatures, (**d**) time, and (**e**) extract concentrations.

Figure 2 shows the XRD diffractograms of the calcined CuO-NiO nanocomposites synthesized under different conditions. Generally, all samples show peaks at 2θ= 37.26°, 43.21°, and 62.74° for the (111), (200), and (220) orientations of nickel oxide, respectively, which are related to the face-centered cubic phase [44,45,54,55]. Copper oxide has peaks at 32.5°, 35.5°, 38.71°, 49.01°, 58.17°, 61.52°, 66.25°, and 68.16° for the (110), (002), (111), (202), (202), (113), (311), and (220) orientations; respectively, which are related to the monoclinic phase [44,45,54,56]. No other phases were detected, confirming the formation of pure NiO and CuO phases within the NiO-CuO nanocomposites without impurities [53]. 

The effect of various synthetic conditions on prepared nanocomposites were studied by following the intensity of normalized XRD peaks, specifically the peaks at 2θ= 35.5°, 37.3°, 38.7°, and 43.2° for CuO (002), NiO (111), CuO (111), and NiO (200) phases, respectively. The rise in the precursor concentration resulted in an increase in the intensity of NiO peaks and a decrease in the intensity of CuO peaks, as shown in Figure 2a. The same observation was noticed for the rise in the pH, with no CuO formed at pH 11, as shown in Figure 2b. Sample 11B showed only the nickel oxide phase. It is reported in the literature that each metal is favorably precipitated at a specific pH, and nickel is precipitated at a higher pH than copper [57]. The increase in the temperature of microwave synthesis resulted in a decrease in the intensity of CuO peaks and induced grain growth of NiO as the intensity of NiO (111) peak increased while the intensity of NiO (200) peak slightly decreased, as shown in Figure 2c [58]. Figure 2d shows that enhancing the synthesis time resulted in an increase in crystallinity. In Figure 2e, sample WE shows low crystallinity owing to the absence of saponin extract compared to the other samples with different concentrations of the extract. This highlights the role of saponin as a capping agent in improving the crystallinity of samples.

**Figure 3:**
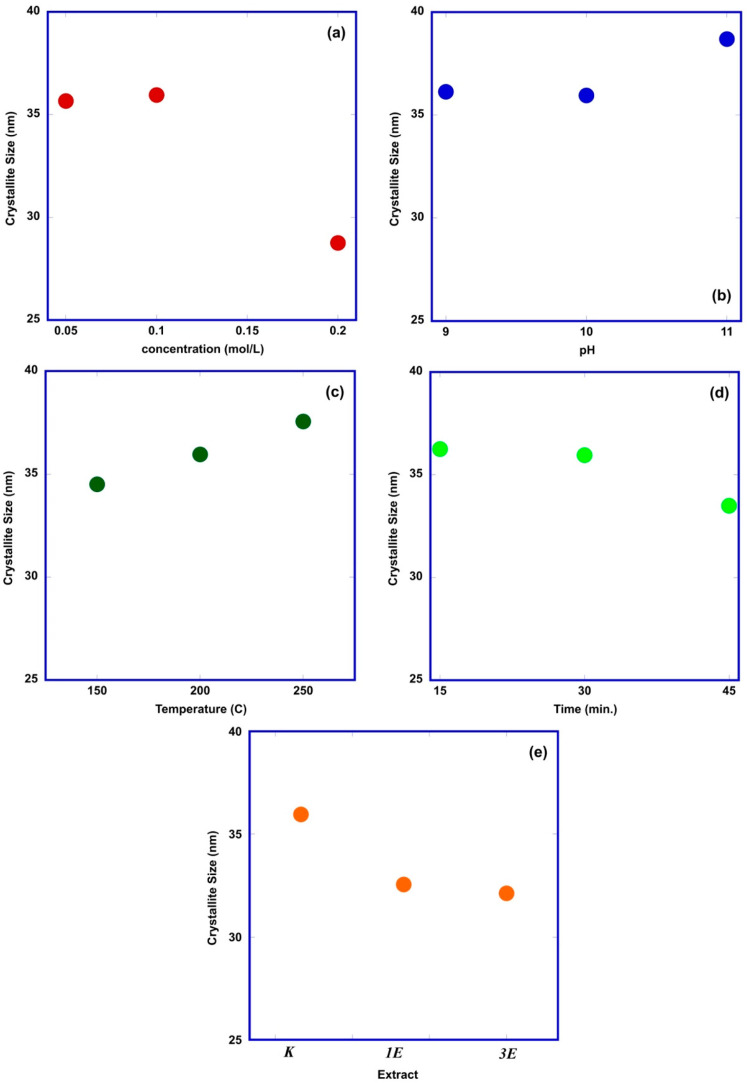
The calculated crystallite size of CuO-NiO nanocomposites at different synthetic conditions: (**a**) salt concentrations, (**b**) pH, (**c**) temperatures, (**d**) time, and (**e**) extract concentrations.

Figure 3a shows the crystallite size of samples 0.05A, 0.1A, and 0.2A, which are 36 nm, 36 nm, and 29 nm, respectively. Increasing the concentration of metal precursors led to larger crystallite sizes due to the presence of more growth units [M(OH)_2_]^4−^ [59]. A further increase in the metal precursors resulted in more negative ions covering the surface of the growing nuclei, which hindered growth and resulted in a decrease in crystallite size [60]. Figure 3b shows the crystallite sizes of samples 9B, 10B, and 11B, which were 36 nm, 36 nm, and 39 nm, respectively. Figure 3c shows the crystallite sizes of samples 150C, 200C, and 250C, which are 35 nm, 36 nm, and 38 nm, respectively. Figure 3d shows the crystallite sizes of samples 15D, 30D, and 45D, which were 36 nm, 36 nm, and 34 nm, respectively. Figure 3e shows the crystallite size of samples WE, K, 1E, and 3E, which are 29 nm, 36 nm, 33 nm, and 32 nm, respectively. It is worth noting that sample WE has a small crystallite size owing to the lack of crystallinity, as observed in Figure 2e. Comparing sample K and sample 1E, the crystallite size decreases from 36 to 33 nm because of the increase in the extract volume from 20 mL to 40 mL from the extract prepared by dissolving 10 g of saponin powder in 100 mL of water. Using 30 mL from the extract prepared by dissolving 20 g of saponin powder in 100 mL of water resulted in a crystallite size of 32 nm, which is close to the size of sample K.

### 3.3. UV-Visible Spectroscopy

**Figure 4:**
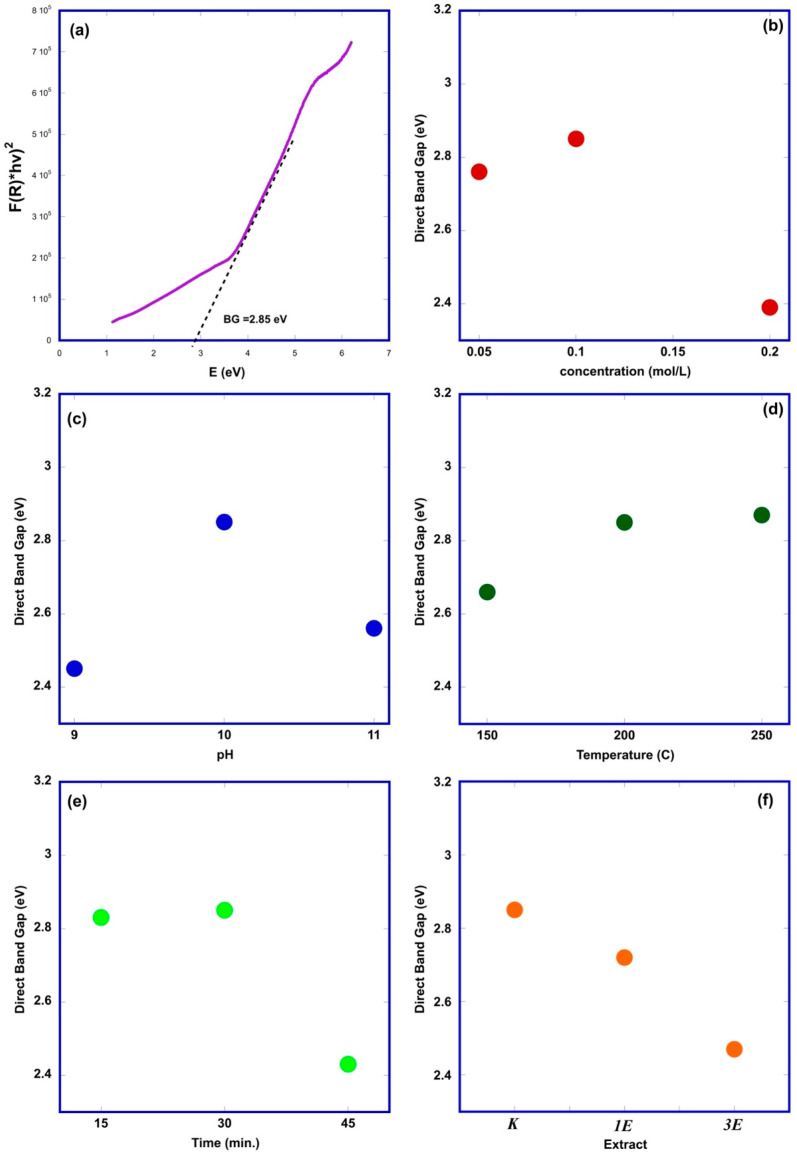
(**a**) Tauc’s plot of sample 0.1A and (**b**–**f**) values of the direct band gap of samples synthesized under different synthetic conditions.

The calculated direct band gap of sample 0.1A is 2.85 eV, as represented in Figure 4a, which is an example of Tauc’s plot. In Tauc’s plot, *hυ* is plotted on the x-axis against (α *hυ*)^2^ on the y-axis, where the intersection of the figure with the x-axis gives the value of the direct band gap. The direct band gaps of the 0.05, 0.1, and 0.2A samples were 2.76 eV, 2.85 eV, and 2.39 eV, respectively, as shown in Figure 4b. The direct band gaps of the 9B, 10B, and 11B samples were 2.45 eV, 2.85 eV, and 2.56 eV, respectively, as displayed in Figure 4c. The direct band gap of the 150C, 200C, and 250C samples are 2.66 eV, 2.85 eV, and 2.87 eV, respectively, as shown in Figure 4d. The direct band gaps of 15D, 30D, and 45D samples are 2.83 eV, 2.85 eV, and 2.43 eV, respectively, as shown in Figure 4e. The direct band gaps of WE, K, 1E, and 3E samples are 3.17 eV, 2.85 eV, 2.72 eV, and 2.47 eV, respectively, as shown in Figure 4f. Overall, the direct band gaps of the samples prepared in this work were in the range of 2.39–3.17 eV. The observed band gaps fall in the visible light range (1.65–3.1 eV), which means that the prepared composites are capable of absorbing and interacting with visible light. This range is favorable for applications in photocatalytic reactions, solar cells, optical devices, sensors, and optoelectronics [38,61,62,63].

The reported Eg of pure CuO and NiO pristine nanoparticles are 1.2–2 eV and 3.4–4.0 eV, respectively, and the Eg of CuO-NiO nanocomposite is 2.84 eV [4,7]. It has been reported that the band gap of a composite is between the band gaps of the components of composites. Comparing the crystallite sizes with band gaps, there is a direct relationship between the crystallite size and band gap. For example, the crystallite sizes of samples 0.05A, 0.1A, and 0.2A are 35.66 nm, 35.95 nm, and 28.75 nm, respectively, and the direct band gaps of the same samples are 2.76 eV, 2.85 eV, and 2.39 eV, respectively. Indeed, there is a correlation between crystallite size and bandgap [64]. H. Weldekirstos et al. prepared CuO-NiO nanocomposites via simple precipitation in the presence of CTAB surfactant, and the band gap calculated with Tauc’s plot was 3.25 eV [38]. S. Anitha et al. measured the band gap using UV-Vis, and it was 2.84 eV. They mentioned that the electronic structure of NiO has been modified through CuO in the way of transitions from the valence band of CuO to the conduction band of NiO, which results in narrowing its band gap [7]. 

### 3.4. Microstructure Characterization

**Figure 5:**
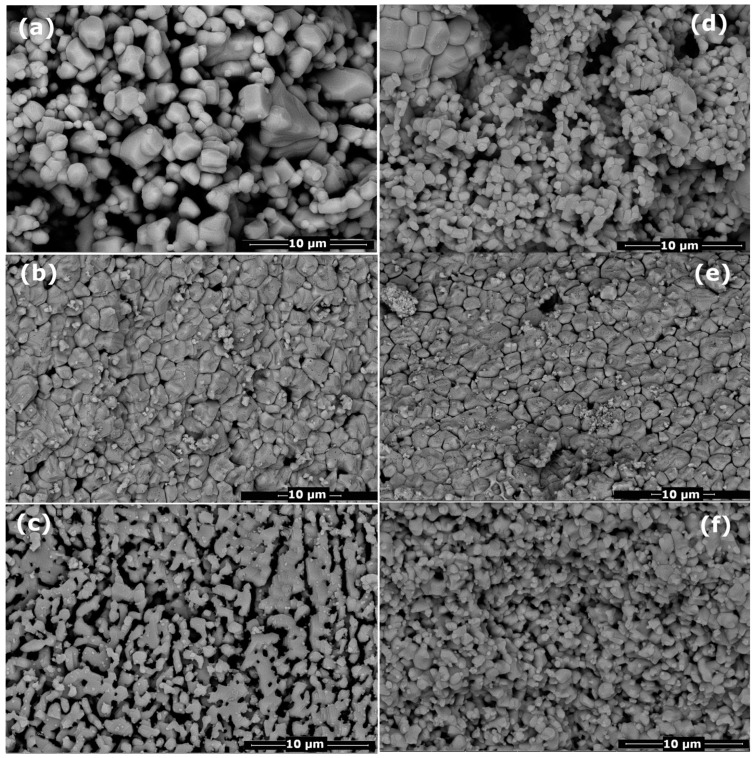
SEM images of samples (**a**) 0.05A, (**b**) 0.1A, (**c**) 0.2A, (**d**) 9B, (**e**) 10B, and (**f**) 11B.

**Figure 6:**
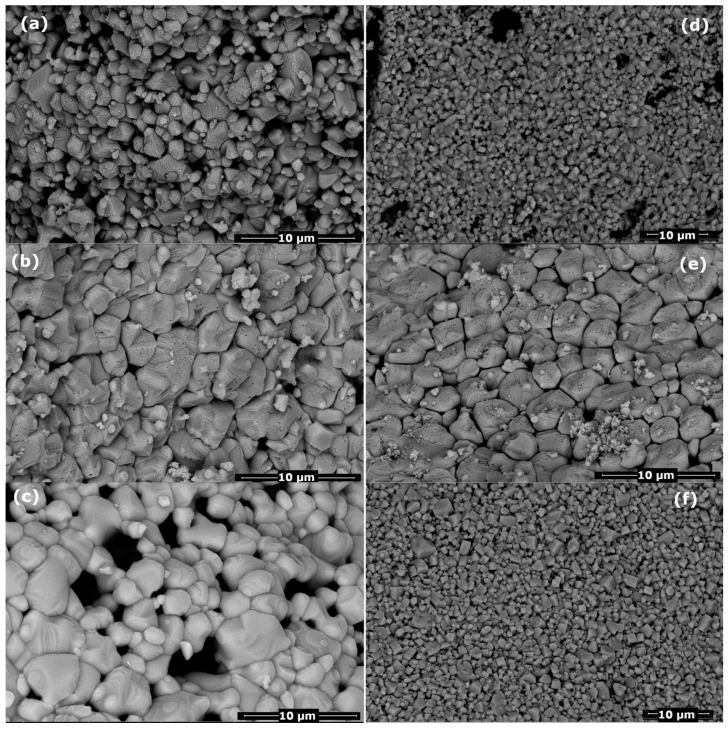
SEM images of samples (**a**) 150C, (**b**) 200C, (**c**) 250C, (**d**) 15D, (**e**) 30D, and (**f**) 45D.

**Figure 7:**
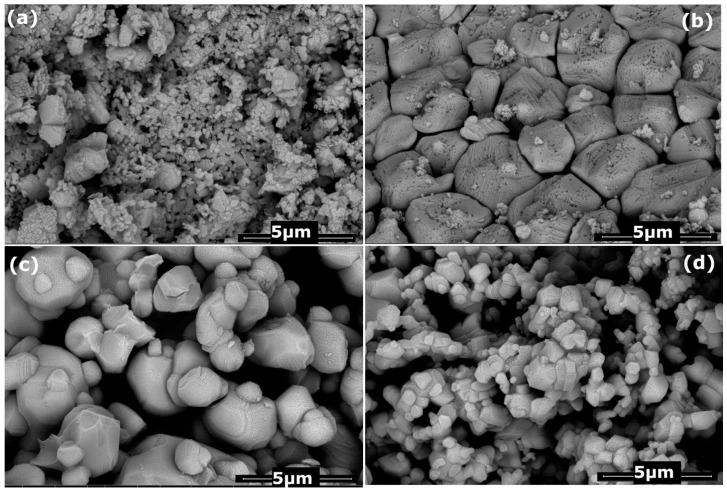
SEM images of samples (**a**) WE, (**b**) K, (**c**) 1E, and (**d**) 3E.

SEM characterization provided valuable insights into the morphologies of the CuO-NiO nanocomposites. Generally, SEM images reveal homogenous and well-dispersed particles. Figure 5a–c shows the concentration effect of the metal precursors on the morphological features of the CuO-NiO nanocomposites. Samples 0.05A, 0.1A, and 0.2A show smooth polygonal, porous polygonal, and worm gear shapes, respectively. The effect of pH during the synthesis process on the morphological features of the CuO-NiO nanocomposites was also investigated using SEM, as shown in Figure 5d–f. Samples 9B, 10B, and 11B show spherical, porous polygonal, and spherical shapes, respectively. The impact of temperature during microwave–hydrothermal synthesis on the morphological characteristics of CuO-NiO nanocomposites was additionally researched using SEM, as shown in Figure 6a–c. Samples 150C, 200C, and 250C show the morphology of smooth polygonal, porous polygonal, and larger smooth polygonal shapes, respectively. The effect of time during the synthesis process on the morphological features of the CuO-NiO nanocomposites was also investigated using SEM, as shown in Figure 6d–f. The effect of the extract concentration on the morphological characteristics of the CuO-NiO nanocomposites is shown in Figure 7a–d. When CuO-NiO was prepared without extract, its morphology was inhomogeneous and highly agglomerated, thus reflecting the importance of using saponin as a capping agent. Samples K, 1E, and 3E show a polygonal shape with a decrease in size as the extract concentration increases. SEM images show the size of agglomerated particles in a micrometer range, while TEM images (will be discussed below) show the size of primary particles in a nanometer range.

**Figure 8:**
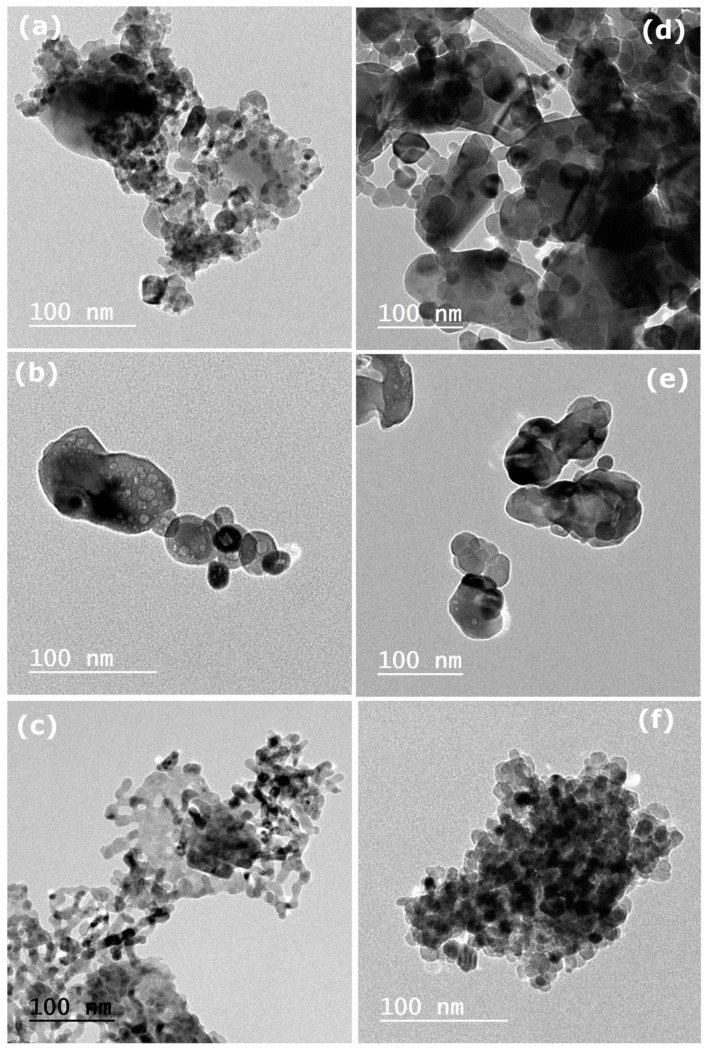
TEM images of samples (**a**) 0.05A, (**b**) 0.1A, (**c**) 0.2A, (**d**) 9B, (**e**) 10B, and (**f**) 11B.

Generally, samples show well-dispersed spherical and quasi-spherical nanoparticles with different sizes based on the synthesis conditions when characterized via TEM. When the concentration of metal precursors is 0.05 M (Figure 8a), the image shows a bimodal size distribution with small spherical nanoparticles anchored on large quasi-spherical sheets. Increasing the concentration to 0.1 M (Figure 8b) results in larger nanoparticles with more homogenous size distribution and pores. Further increase in the concentration to 0.2 M (Figure 8c) results in fused nanoparticles with trapped intrapores.

When the pH of synthesis is 9 (Figure 8d), the image shows a bimodal size distribution with small spherical nanoparticles anchored on large quasi-spherical sheets. Increasing the pH to 10 (Figure 8e) results in a more homogenous size distribution. The increase in pH to 11 (Figure 8f) results in the formation of smaller particle sizes with more agglomeration, which can be explained, as mentioned previously, by restricted adsorption of the negatively charged complex [M (OH)_4_]^2−^ on the negative surface of MO.

**Figure 9:**
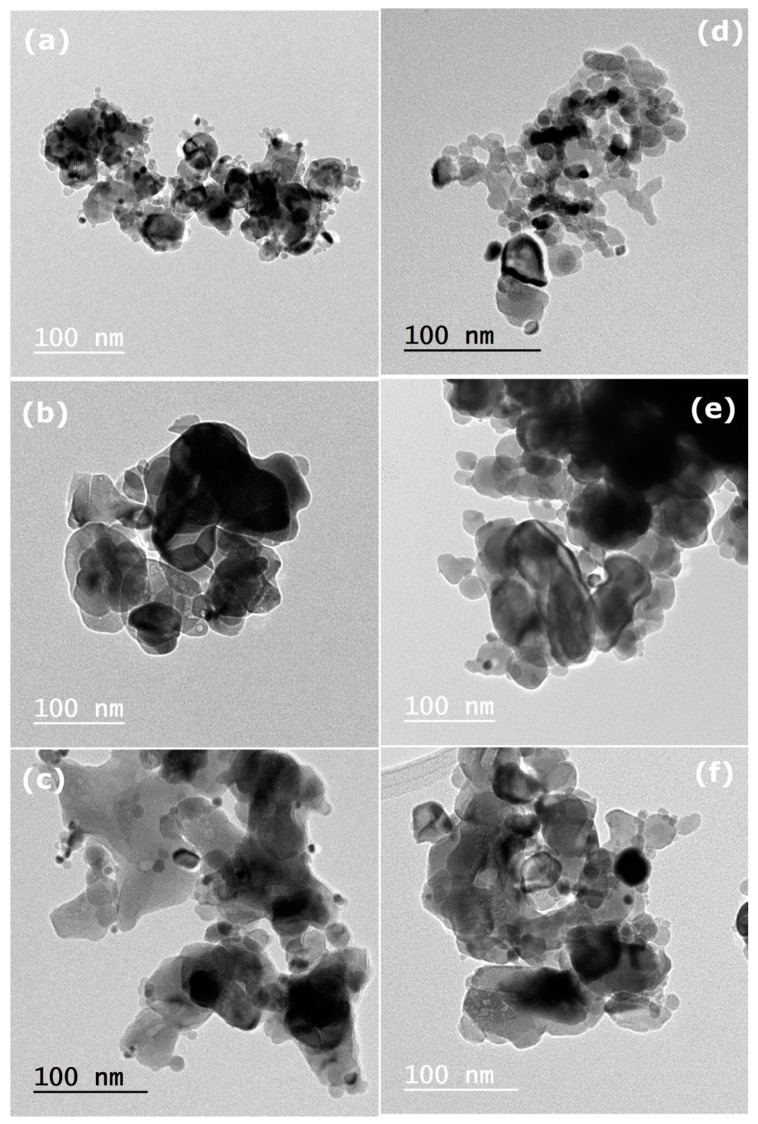
TEM images of samples (**a**) 150C, (**b**) 200C, (**c**) 250C, (**d**) 15D, (**e**) 30D, and (**f**) 45D.

**Figure 10:**
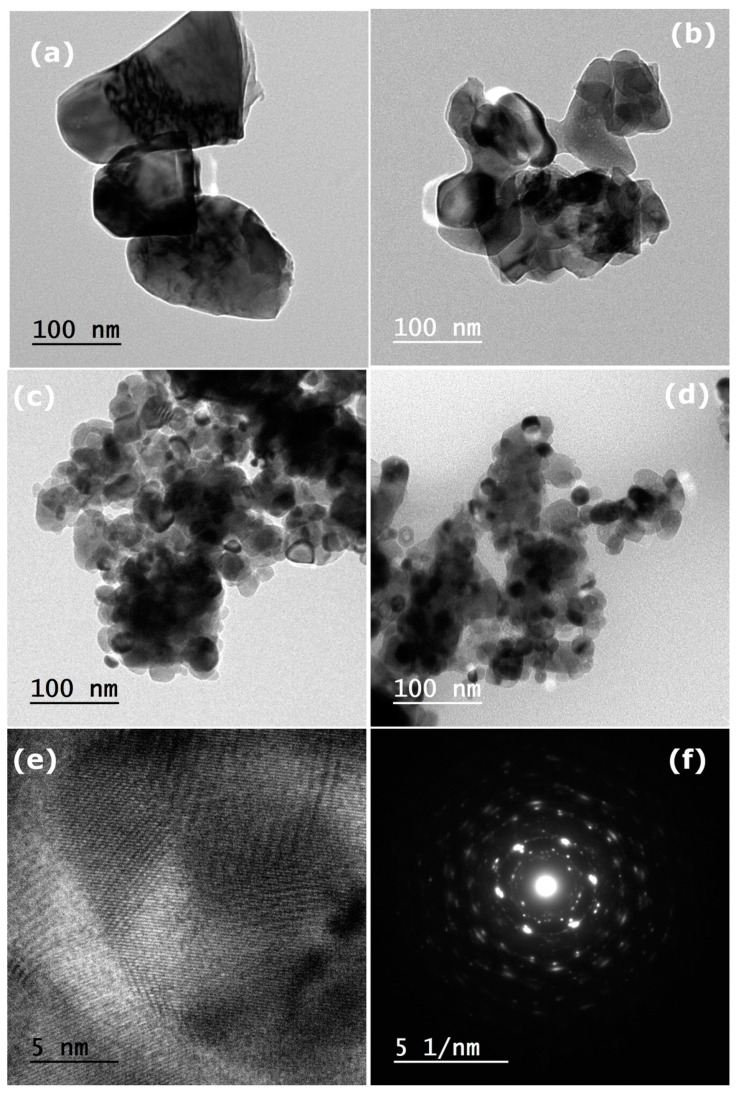
TEM images of samples (**a**) WE, (**b**) K, (**c**) 1E, (**d**) 3E, (**e**) HR-TEM image, and (**f**) SAED image.

Increasing the temperature and time of microwave treatment results in an increasing growth rate and increases the particle size, as depicted in Figure 9. The effect of using extract is obvious when comparing sample WE (Figure 10a) with other samples, as shown in Figure 10. Sample WE showed larger particle sizes that exceeded 100 nm, while the particle sizes were approximately 40 nm, 35 nm, and 32 nm for samples K, 1E, and 3E samples, as shown in Figure 10b, c, and d, respectively. Figure 10e shows the HR-TEM of sample K with parallel lattice fringes indicating good crystallinity. Figure 10f shows bright spots superimposed on ring patterns, confirming the polycrystalline nature of the nanocomposites. Moreover, the crystallite size calculated from XRD is in the range of 29–39 nm, which is smaller than the size observed in TEM, indicating the polycrystallinity of prepared samples [65,66,67].

Based on the diverse outcomes, a mechanism for nanocomposite formation can be suggested. Initially, the metal salt precursor is mixed with the extract and precipitated using ammonium hydroxide to form a mixture of metal hydroxide [Cu/Ni(OH)_2_]. The particles of Cu/Ni(OH)_2_ are subsequently subjected to controlled thermal dehydration in an autoclave at specific temperatures, leading to the formation of the final CuO-NiO nanocomposites. The dimensions, size, and quality of the CuO-NiO nanocomposites are primarily governed by experimental factors such as the concentration of the metal precursor, pH, growth duration, and growth temperature [68]. This method employs environmentally friendly reaction conditions in its green synthesis process, such as using water as a solvent, applying low reaction temperatures, and the absence of toxic chemicals or hazardous solvents. This approach not only reduces the environmental footprint of the synthesis process but also ensures the production of a nanocomposite with desirable properties. This method utilizes microwave radiation to generate localized hot spots, promoting the nucleation and growth of CuO and NiO nanoparticles.

## 4. Conclusions

The formation of the CuO-NiO nanocomposite through microwave–hydrothermal-assisted green synthesis offers several advantages. First, the use of microwave radiation enables rapid and efficient heating, leading to a shorter synthesis time compared to conventional methods. Second, the hydrothermal conditions provide a controlled environment for the nucleation and growth of CuO and NiO nanoparticles, resulting in a well-defined and highly uniform nanocomposite structure. Third, the green synthesis principles employed in this method minimize the use of toxic chemicals and hazardous solvents, making it a more sustainable and environmentally friendly approach to nanocomposite synthesis. Furthermore, the CuO-NiO nanocomposite synthesized via microwave–hydrothermal assisted green synthesis exhibits improved properties compared to its individual components.

## Figures and Tables

**Table 1:** Sample names and corresponding conditions.

	**Effect of Metal Precursor Concentration**				
Sample Code	A: Conc.	B: pH	C: Temp.	D: Time	E: Extract Vol. (g of saponin/Vol. Water)
0.05A	0.05	10	200	30	20 mL (1 g/10 mL)
0.1A	0.1	10	200	30	20 mL (1 g/10 mL)
0.2A	0.2	10	200	30	20 mL (1 g/10 mL)
	**Effect of pH**				
9B	0.1	9	200	30	20 mL (1 g/10 mL)
10B	0.1	10	200	30	20 mL (1 g/10 mL)
11B	0.1	11	200	30	20 mL (1 g/10 mL)
	**Effect of Temperature**				
150C	0.1	10	150	30	20 mL (1 g/10 mL)
200C	0.1	10	200	30	20 mL (1 g/10 mL)
250C	0.1	10	250	30	20 mL (1 g/10 mL)
	**Effect of Time**				
15D	0.1	10	200	15	20 mL (1 g/10 mL)
30D	0.1	10	200	30	20 mL (1 g/10 mL)
45D	0.1	10	200	45	20 mL (1 g/10 mL)
	**Effect of Extract Concentration**				
WE	0.1	10	200	30	*-*
K	0.1	10	200	30	20 mL (1 g/10 mL) = 2 g
1E	0.1	10	200	30	40 mL (1 g/10 mL) = 4 g
3E	0.1	10	200	30	30 L (2 g/10 mL) = 6 g

## Data Availability

Data only available upon request from the corresponding authors.
